# The doses of plasmid backbone plays a major role in determining the HBV clearance in hydrodynamic injection mouse model

**DOI:** 10.1186/s12985-018-1002-y

**Published:** 2018-05-21

**Authors:** Xian Wang, Jianmin Zhu, Yong Zhang, Yue Li, Tai Ma, Qun Li, Jiegou Xu, Long Xu

**Affiliations:** 1 School of Basic Medical Sciences, Anhui Medical University, 81#Mei Shan Road, Hefei, 230032 Anhui China; 2 Pediatric Translational Medicine Institute, Shanghai Children’ s Medical Center, Shanghai Jiaotong University School of Medicine, Shanghai, China; 3 Department of Pathology, The Second Affiliated Hospital of Anhui Medical University, Hefei, Anhui China; 4 Department of Spine Surgery, The First Affiliated Hospital of Anhui Medical University, Hefei, Anhui China

**Keywords:** Plasmid backbone, Hepatitis B virus, Hydrodynamic injection, HBcAg, Mouse model

## Abstract

**Background:**

Hepatitis B virus (HBV) chronically infects approximately 350 million people worldwide, causing a major risk of liver disease and hepatocellular carcinoma (HCC). Many mouse models have been tried to establish HBV infection through injection with various HBV-containing plasmids. However, it is not well understood that different plasmids, all of which contain the similar HBV genome, even the same plasmids with different dose, results in opposite immune responses toward HBV.

**Methods:**

In this study, we investigated the role of HBV-containing plasmid backbones and the HBcAg in determining the HBV persistence. C57BL/6 mice were injected hydrodynamically with 6 μg or 20 μg of WT pAAV/HBV1.2 plasmid, e/core-null pAAV/HBV1.2 plasmid, or none-HBV genome pAAV/control plasmid. Serum levels of HBV-related markers were measured by quantitative immunoradiometric assay (IRMA). Liver HBcAg expression was detected by immunohistochemical staining. The mRNA levels of cytokines and Th1-related immune factors were quantified by qRT-PCR.

**Results:**

All mice injected with 6 μg of the pAAV/HBV1.2 plasmid shows HBsAg positive at week 6 after hydrodynamic injection (AHI) as previously investigated. However, the mice injected with 20 μg pAAV/HBV1.2 or 6μgpAAV/HBV1.2 plus 14μgpAAV/control plasmid results in HBV clearance within 4 weeks AHI, indicating the anti-HBV activity is induced by 20 μg plasmid DNA, but not by the inserted viral genome. This anti-HBV activity is independent of HBcAg and Toll like receptor (TLR) signaling pathway, since the lack of HBcAg in pAAV/HBV1.2 plasmid or stimulation with TLRs agonists does not influence the kinetics of serum HBsAg in mice. The mRNA levels of t-bet and cxcr3 were dramatically up-regulated in the liver of the mice injected with 20 μg plasmid DNA.

**Conclusion:**

Our studies demonstrate that plasmid backbones are responsible for modulating immune responses to determine HBV persistence or clearance in our HBV mouse model by hydrodynamic injection of HBV-containing plasmid, and Th1 cells play key roles on HBV clearance.

## Background

Hepatitis B virus (HBV), a member of the hepadnavirus group, is a double-stranded DNA virus which replicate by reverse transcription [1]. It causes acute and chronic viral hepatitis which is a major public health problem that affects about 350 million people worldwide. Moreover, 25% persistent HBV sufferers would develop into hepatic cirrhosis and hepatocellular carcinoma [2–4]. However, the precise mechanisms of HBV immunopathogenesis and the virus persistence are not well understood yet.

Natural HBV infection only occurs in human being, chimpanzee and tree shrew [5]. So, lack of suitable mouse models is an obstacle to explore the immunological mechanism of HBV. Although the appearance of HBV-transgenic mice has promoted HBV related research [6], it is not a good model to investigate the immunological mechanisms of HBV because of the central tolerance to HBV-related antigens. To overcome this challenge, many researchers created HBV persistence models in immunocompetent mice through hydrodynamic injection of HBV genome-containing plasmids, by which the injected plasmids could mainly target hepatocytes [7]. In these HBV persistent mice, viral replication intermediates, transcripts, and all HBV related proteins can be detected in the liver tissues for several months. However, hydrodynamic injection of different plasmids, in spite of containing same HBV genome, or the same plasmids at different doses induce totally different immune responses toward HBV and subsequently result in HBV persistence or HBV clearance [8–11]. This opposite results suggest that plasmid backbones other than HBV genome are involved in triggering innate immunity [12], which eventually influence the status of HBV infection.

In the present study, through hydrodynamic injection with pAAV/HBV1.2 plasmid which was widely used to establish HBV-carrier mouse models in our previous studies [13–16], we found that the plasmid-injected mice showed a completely different immune response toward HBV. Six micrograms of the pAAV/HBV1.2 pasmids induced a long-term humoral immune tolerance accompanied by persistent serum HBsAg levels and HBcAg in liver tissue, while 20 μg pAAV/HBV1.2 plasmid resulted in rapid HBV clearance within 4 weeks, along with significantly increased serum anti-HBs antibody levels. Finally, we found the doses of plasmid backbones, but not HBV-related components, played critical roles in determining the HBV clearance in our mouse model.

## Methods

### Animals

Male C57BL/6 mice (5–6 weeks old) were purchased from the Shanghai Experimental Animal Center (Shanghai, China). The mice were maintained under specific pathogen-free conditions and used according to the guidelines outlined in the Guide for the Care and Use of Laboratory Animals.

### Plasmid and mouse model

WT pAAV/HBV1.2 and e/core-null pAAV/HBV1.2 plasmid were kindly provided by Dr. Peijer Chen (National Taiwan University). All the plasmids were isolated by using an endotoxin-free midi kit (Qiagen, Inc., Valencia, CA, USA). Hydrodynamic injection of the different plasmids into mice was performed as described [7]. Serum HBsAg, anti-HBs, and anti-HBc antibody levels were determined using commercially available immunoradiometric assay (IRMA) kits (Beijing North Institute of Biological Technology, Beijing, China).

### Immunohistochemistry

Liver samples were fixed in 10% neutral buffered formalin and embedded in paraffin. Liver sections were stained for HBcAg using rabbit antibodies against HBcAg (Dako, Carpinteria, CA) followed by biotinylated anti-rabbit IgG and streptavidin-HRP conjugates (Zhongshan Goldenbridge, Beijing, China). The stains were developed with a DAB kit (Vector Laboratories).

### Reverse transcriptase - PCR

Total RNA was isolated from the liver tissues or liver mononuclear cells (MNCs) at day 3 AHI by TRizol reagent (Invitrogen). Reverse transcription was performed by M-MLV (Invitrogen). Realtime fluorescence quantitative PCR was based on SYBR Premix Ex Tap (TaKaRA), and the reactions were performed with a denaturation step at 94 °C for 20s, annealing at 60 °C for 30s, and extension at 72 °C for 30s for 40 cycles. Results were normalized to GAPDH mRNA expression. PCR primers are as followed:

tlr3, TTGTCTTCTGCACGAACCTG (f) and CGCAACGCAAGGATTTTATT (r);

tlr4, ACCTGGCTGGTTACACGTC (f) and CTGCCAGAGACATTGCAGAA(r);

tlr7, GGAAATTGCCCTCGATGTTA (f) and CAAAAATTTGGCCTCCTCAA (r);

tlr8, GAAGCATTTCGAGCATCTCC (f) and GAAGACGATTTCGCCAAGAG (r);

tlr9, ACTGAGCACCCCTGCTTCTA (f) and AGATTAGTCAGCGGCAGGAA (r);

ifn-α, AGGACAGGAAGGATTTTGG (f) and GCTGCTGATGGAGGTCAT (r);

ifn-β, CACAGCCCTCTCCATCAAC (f) and GCATCTTCTCCGTCATCTCC (r);

ifn-γ, TGCATCTTGGCTTTGCAGCTCT (f) and TGGACCTGTGGGTTGTTGACCT (r);

il-6, ACAACCACGGCCTTCCCTAC (f) and ACAATCAGAATTGCCATTGCAC (r);

il-12, GTGAACCTCACCTGTGACACGC (f) and AATACTTCTCATAGTCCCTTTGG (r);

il-15, CCAACTGGATAGATGTAAGATA (f) and GTCAGGACGTGTTGATGAACAT (r);

tgf-β, GTACAGCAAGGTCCTTGCCCT (f) and TAGTAGACGATGGGCAGTGGC (r);

t-bet, GCCAGGGAACCGCTTATATGTC (f) and CTGTGAGATCATATCCTTGGGCTG (r);

cxcr3, TGTAGCCCTCACCTGCATAGTTGT (f) and GTTGTACTGGCAATGGGTGGCATT (r); gapdh, ACCACAGTCCATGCCATCAC (f) and TCCACCACCCTGTTGCTGTA (r).

f and r mean forward and reverse pimers.

### Statistical analysis

Unpaired two-tailed Student’s *t*-test was used for statistical analyses. Data was expressed as means ± SEM, and data were considered statistically significant when *P* values were < 0.05. Significance was denoted as *P < 0.05, **P < 0.01, and ***P < 0.001.

## Results

### Injection of 20 μg pAAV/HBV1.2 plasmids induced anti-HBV activity in mice model

**Fig. 1:**
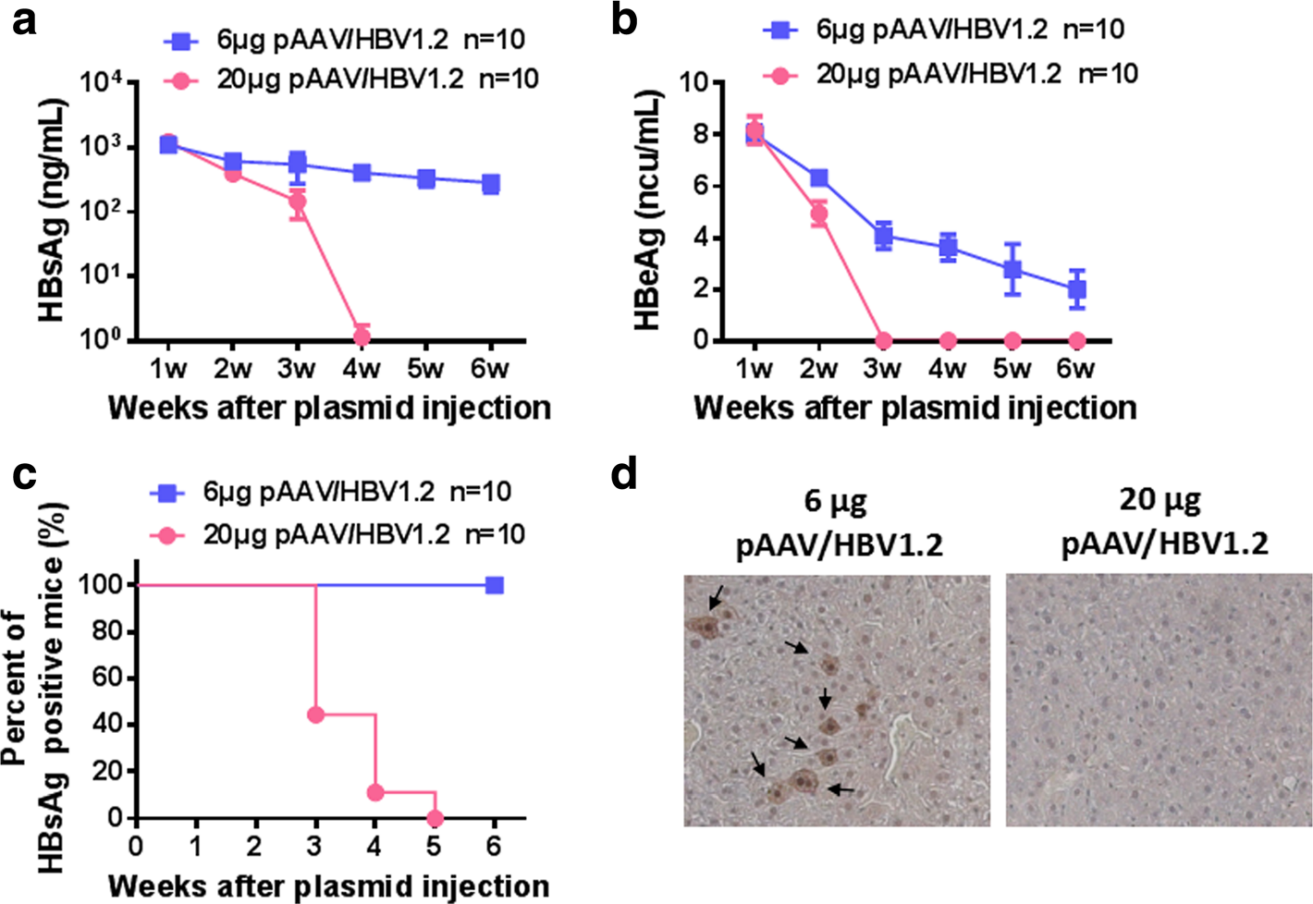
The doses of pAAV/HBV1.2 plasmid determine HBV persistence in C57BL/6 mice. C57BL/6 mice were hydrodynamically injected with 6 μg or 20 μg pAAV/HBV1.2 plasmid. Serum levels of HBsAg (**a**) and HBeAg (**b**) were measured at indicated time points. **c** Positive rate of serum HBsAg after HBV plasmid injection. **d** Immunohistochemical staining for HBcAg in liver sections of mice at week 6 after injection with 6 μg or 20 μg pAAV/HBV1.2 plasmid. (original magnification: 100×). Results represent 2 independent experiments (*n* = 10/group)

Our previous studies have revealed that hydrodynamic injection of 6 μg pAAV/HBV1.2 could establish long-term HBsAg-persistent mice and maintain the tolerant state via induction of HBV specific Tr1 like cells [13, 17]. In this study, C57BL/6 mice were injected with 6 μg or 20 μg pAAV/HBV1.2 plasmids. In the 20 μg group, the serum levels of HBsAg and HBeAg dropped quickly and all the mice were HBsAg negative at 5 weeks post injection (wpi) (Fig. 1a-c), indicating the induction of anti-HBV immunity in theses mice. However, in the 6 μg group, the HBsAg and HBeAg level declined much more slowly (Fig. 1a, b), and all mice were still HBsAg positive at 6 wpi (Fig. 1c). Also, 80% of the mice injected with 20 μg pAAV/HBV1.2 plasmids produced anti-HBs antibody in the serum, while only 25% of those receiving 6 μg pAAV/HBV1.2 plasmid were anti-HBs positive at week 11 AHI (Table 1). In addition, cytoplasmic and nucleic HBcAg was observed in the liver of 6 μg group, but not in that of the 20 μg group, at 6 wpi (Fig. 1d). Overall, hydrodynamic injection of 6 μg pAAV/HBV1.2 induced immune tolerance to keep HBV persistence, while injection of 20 μg pAAV/HBV1.2 triggerd anti-HBV immune responses leading to HBV clearance.

**Table 1:** Appearance of anti-HBs antibody at indicated time points AHI with different HBV plasmids DNA

HBV plasmid	Week 1–3	Week 4	Week 5	Week 11
6 μg WT pAAV/HBV1.2	0/20	0/20	0/20	5/20
20 μg WT pAAV/HBV1.2	0/20	0/20	4/20	16/20
20 μg e/core-null pAAV/HBV1.2	0/10	1/10	3/10	10/10
6 μg WT pAAV/HBV1.2 plus 14 μg pAAV/Control	0/20	1/20	5/20	18/20

The number of mice in each group positive for anti-HBs antibody

### Anti-HBV activity triggered by 20 μg pAAV/HBV1.2 plasmids did not break HBV tolerance induced by 6 μg pAAV/HBV1.2 plasmids

**Fig. 2:**
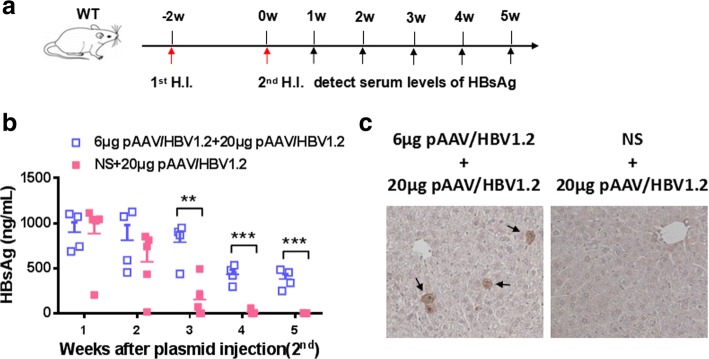
HBV tolerant mice still keep HBV persistence after injection with 20 μg pAAV/HBV1.2. **a** WT mice were hydrodynamically injected with NS or 6 μg pAAV/HBV1.2 plasmid, and then the mice received second injection with 20 μg pAAV/HBV1.2 plasmid. Serum levels of HBsAg were examined at indicated time points. The serum levels of HBsAg (**b**) and liver HBcAg expression (**c**) were shown. Results represent 2 independent experiments (*n* = 5/group)

To test whether anti-HBV activity triggered by 20 μg pAAV/HBV1.2 plasmids, as well as IL-12-based vaccination, could reverse 6 μg pAAV/HBV1.2 plasmid -induced HBV persistence [18], HBV-persistent mice established by prior injection with 6 μg pAAV/HBV1.2plasmid were received second hydrodynamic injection with 20 μg pAAV/HBV1.2plasmid (Fig. 2a). After the second injection, serum levels of HBsAg decreased significantly in normal saline (NS)-pretreated mice and became negative at 5 wpi, while 100% of HBV-persistent mice still kept HBsAg positive in serum, as well as HBcAg in liver section, at 5 wpi (Fig. 2b-c).

### HBV clearance triggered by 20 μg pAAV/HBV1.2 plasmid was independent on HBcAg

**Fig. 3:**
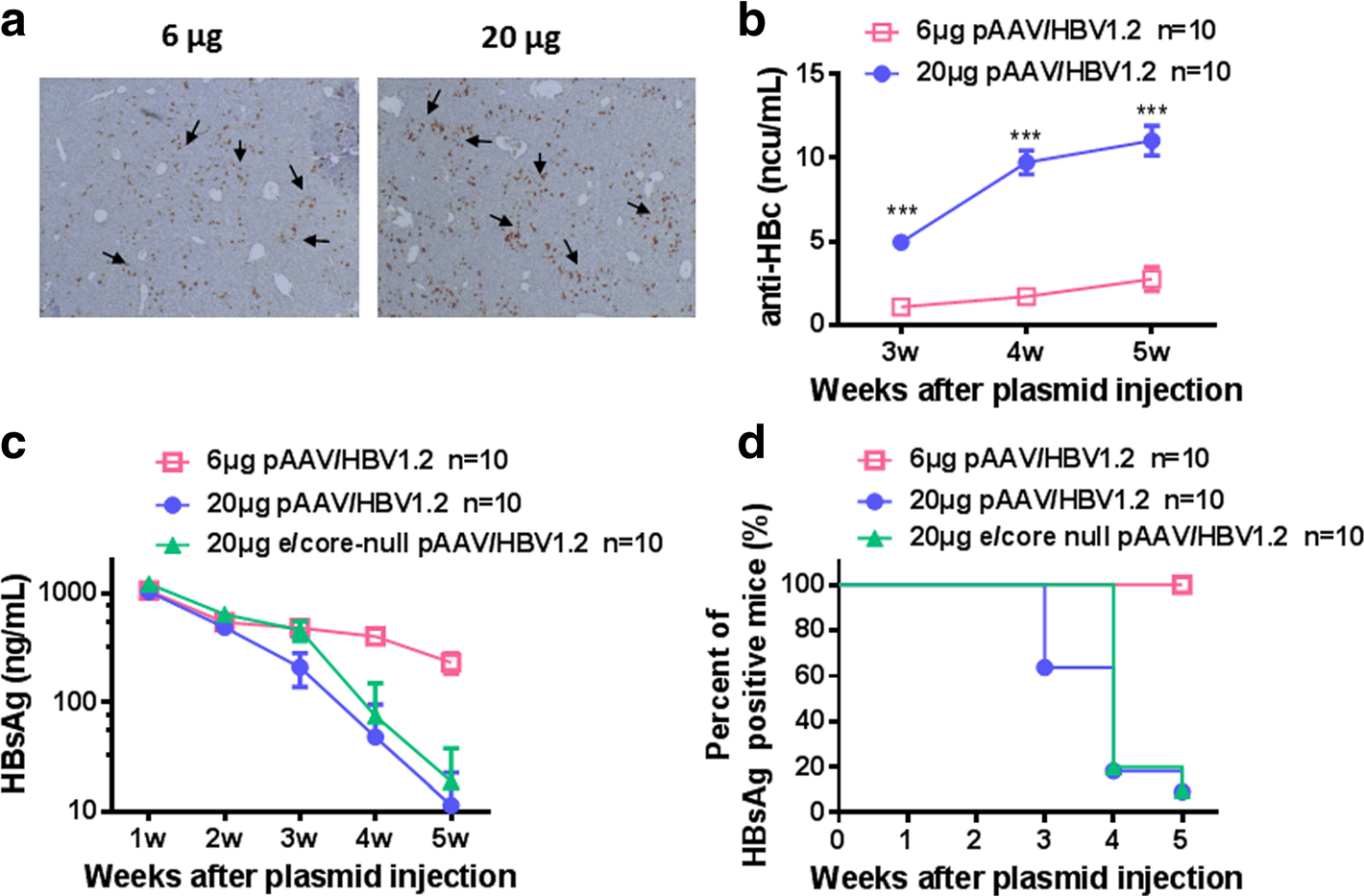
HBV clearance still occurs in 20 μg e/core-null pAAV/HBV1.2 plasmid-injected mice. WT mice were hydrodynamically injected with 6 μg or 20 μg pAAV/HBV1.2 plasmid. Liver HBcAg expression (**a**) was detected at day 3 AHI. (original magnification: 40×) (**b**) serum levels of anti-HBc was measured at indicated mice. **c**, **d** C57BL/6 mice were hydrodynamically injected with various plasmids as shown. Serum levels of HBsAg were measured at indicated time points. **d** Positive rate of serum HBsAg after HBV plasmid injection

It was reported that HBcAg played an important role in inducing anti-HBV immunity [19]. Next we tested whether the anti-HBV activity triggered by 20 μg pAAV/HBV1.2 plasmid was dependent on elevated HBcAg expression, which might be due to the higher concentration of injected plasmids. We indeed found that the percentage of HBcAg positive hepatocytes was much higher in the 20 μg group than those in the 6 μg group (Fig. 3a). Consistently, serum level of anti-HBc antibody was increased significantly in the 20 μg group (Fig. 3b). To further address this point, e/core-null pAAV/HBV1.2 plasmids that was lack of e/core gene was tested. Surprisingly, the serum levels and the persistence rates of HBsAg in mice receiving 20 μg e/core-null mutant plasmids were entirely similar to those of the mice receiving 20 μg WT pAAV/HBV1.2 plasmids (Fig. 3c, d). In addition, the serum levels of anti-HBs displayed the similar kinetics at different time points between these two groups (Table 1). These data indicated that HBV clearance caused by injection of 20 μg pAAV/HBV1.2 plasmids was not associated with HBcAg.

### HBV clearance triggered by 20 μg pAAV/HBV1.2 plasmid was dependent on the doses of plasmid backbone

**Fig. 4:**
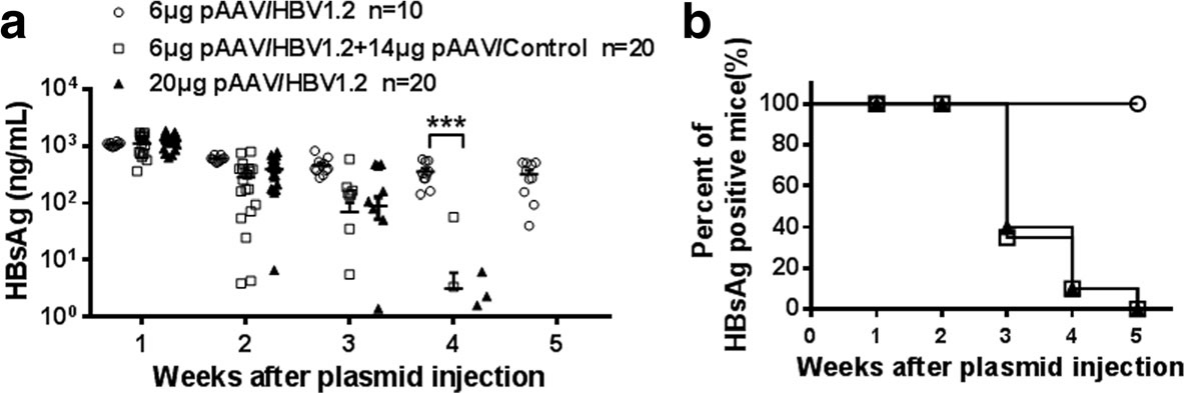
The doses of plasmid backbone are critical for HBV persistence. WT mice were hydrodynamically injected with 6 μg pAAV/HBV1.2, 6 μg pAAV/HBV1.2 plus 14 μg pAAV/control, and 20 μg pAAV/HBV1.2 respectively. Serum levels of HBsAg (**a**) and positive rate of serum HBsAg (**b**) were shown at indicated time points

Another possibility counting for HBV clearance or persistence is the dose of the injected plasmid DNA, which was reported to be recognized by various pattern recognition receptors (PRRs) and eventually trigger innate immune responses [20]. To test this possibility, pAAV/HBV1.2 plasmids and the control plasmids lacking the HBV genome were injected into C57BL/6 mice. 6 μg pAAV/HBV1.2 plasmids were applied in group A; 6 μg pAAV/HBV1.2 plus14μg the control plasmids in group B; and 20μg pAAV/HBV1.2 plasmids in group C, respectively. The serum HBsAg levels of the group B dropped as quickly as the group C, and revealed similar kinetics of serum HBsAg and anti-HBs at different time points. However, these parameters in the group A were totally different from those in the group B, although the groupA and B received the same doses of 6 μg HBV genome DNA (Fig. 4a, b; Table 1). These data indicated that anti-HBV activity was determined by the dose of plasmid backbone, but not HBV-related proteins in our mouse model.

**Fig. 5:**
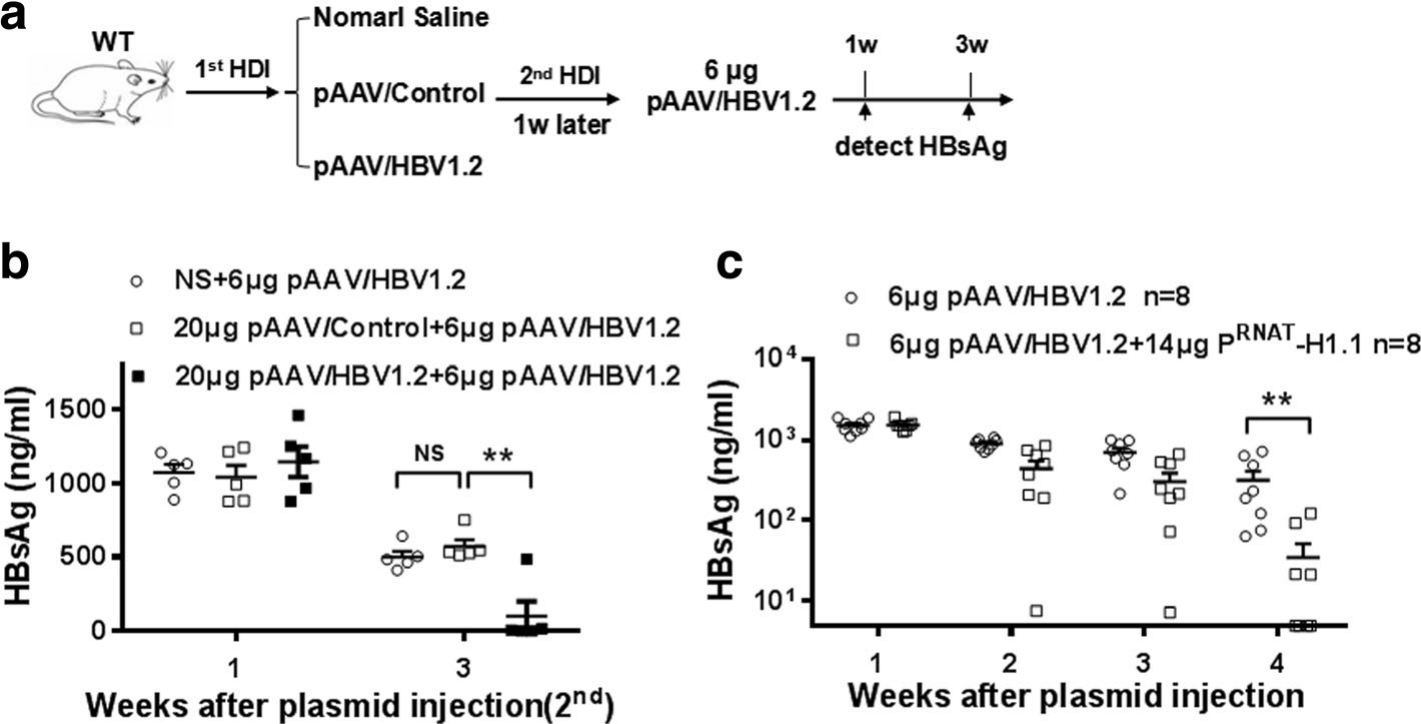
The plasmid backbone causes HBV clearance in unspecific manner. **a** WT mice received first hydrodynamic injection with NS, 20 μg pAAV/Control plasmid, or 20 μg pAAV/HBV1.2 plasmid, and then followed by second injection with 6 μg pAAV/HBV1.2 plasmid 1 week later. **b** Serum levels of HBsAg were determined at week 1 and 3 AHI. **c** Serum levels of HBsAg were detected at the indicated time points after hydrodynamical injection with 6 μg of pAAV/HBV1.2 mixing with or without 14 μg pRNT-H1.1 plasmids respectively. Results represent 2 independent experiments (*n* = 5–8/group)

To test whether 20 μg pAAV/HBV1.2 plasmids-caused HBV clearance was mediated by immune response toward the plasmid backbone or toward HBV-related antigens, we injected 20 μg the control or 20 μg pAAV/HBV1.2 plasmids into mice 1 week prior to injection of 6 μg pAAV/HBV1.2 (Fig. 5a). Three weeks later, the mice pre-treated with 20 μg of the control plasmids still kept HBV persistence, similar to those pre-treated with NS. However, 80% of mice pre-treated with 20 μg pAAV/HBV1.2 developed anti-HBV activity and eliminated the serum HBsAg (Fig. 5b). These data suggested that the plasmid backbone, which must been injected together with 6 μg pAAV/HBV1.2, had an adjuvant effect to induce the immune activity toward HBsAg in a nonspecific manner. To further address this, we found co-injection of 6 μg pAAV/HBV1.2 and 14 μg HBV-irrelevant pRNT-H1.1 plasmid could also lead to the significant decrease of HBsAg levels at week 4 AHI (Fig. 5c).

### The mRNA levels of toll like receptors (TLRs) and Th1-related immune factor at day 3 AHI

**Fig. 6:**
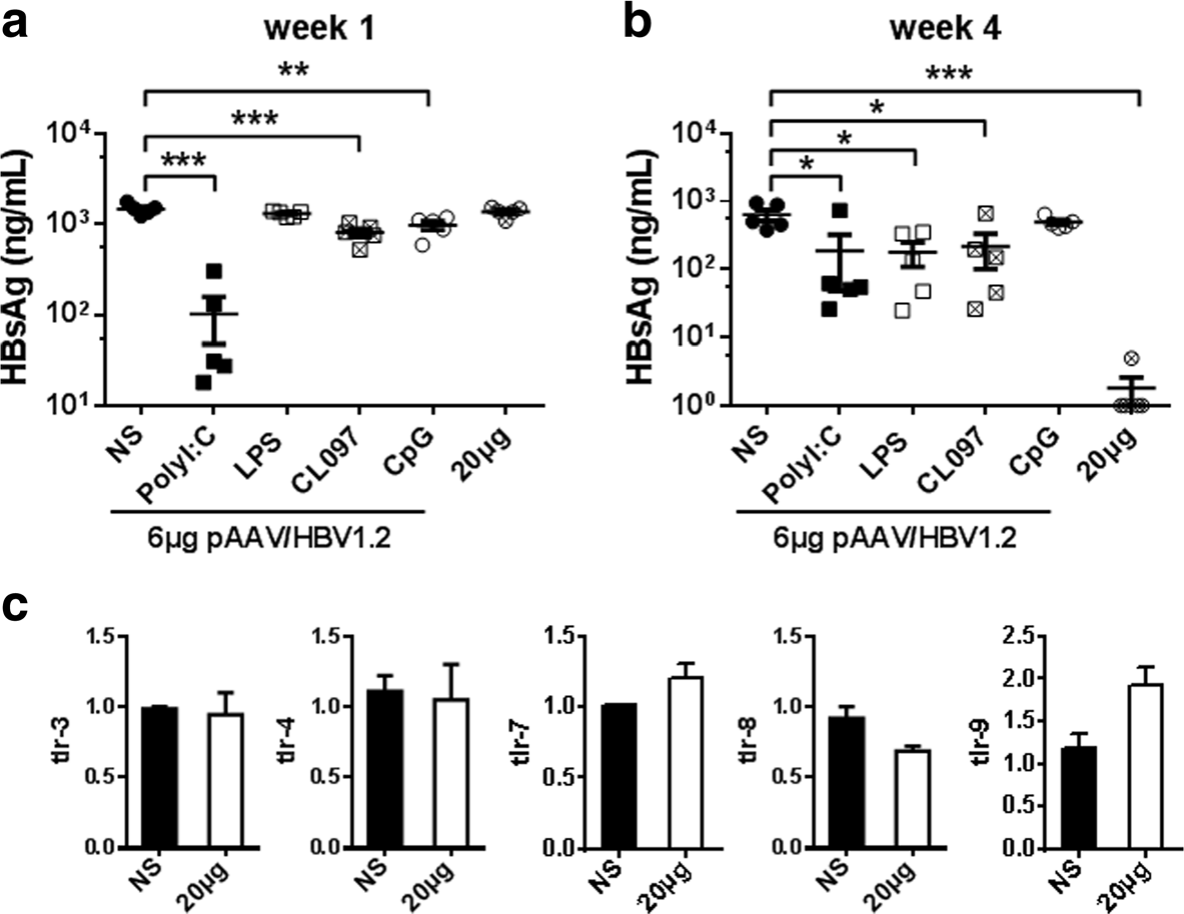
The TLRs signaling pathway is not critical for HBV clearance in high dose HBV-plasmid injection. C57BL/6 mice were injected with 6 μg pAAV/HBV1.2 mixing with different TLR activators, including: 10 μg of poly I:C, LPS, CL097 and CpG, respectively. Serum levels of HBsAg were measured at week 1 (**a**) and week 4 (**b**) AHI. **c** The mRNA levels of TLRs in liver tissue was detected by real time PCR at day 3 after injection of NS or 20 μg pAAV/control plasmid. Results represent 2 independent experiments (*n* = 5/group)

To explore a possible link between 20 μg plasmid DNA caused HBV clearance and TLRs signaling pathways [21, 22], serum levels of HBsAg were monitored from mice receiving 6 μg pAAV/HBV1.2 mixed with agonists of TLR3, TLR4, TLR7/8, and TLR9 respectively. All these mice have shown the decreased levels of HBsAg at week 1 or week 4, but none of mice were HBsAg negative at week 4 AHI (Fig. 6a-b). Also, the relative mRNA levels of TLR3, TLR4, TLR7, TLR8, or TLR9 kept unaltered in liver tissue of mice receiving NS or 20 μg of the control plasmid (Fig. 6c), suggesting the TLRs signaling pathway did not account for HBV elimination in this model.

**Fig. 7:**
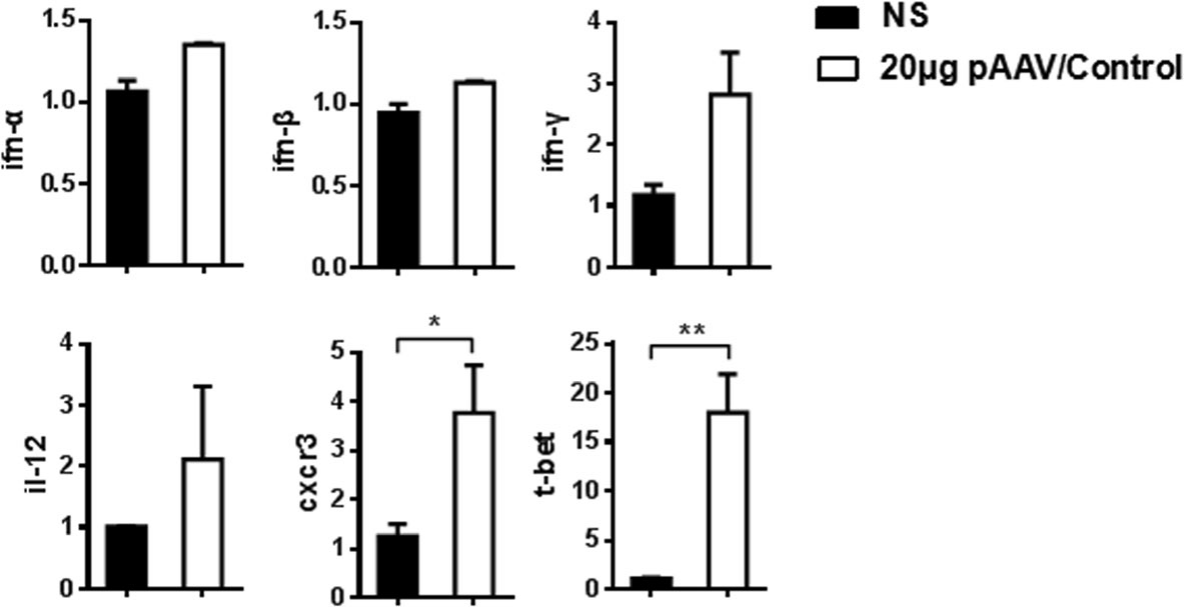
Relative mRNA expression. Relative amount of mRNA in liver MNCs was detected by real time PCR at day 3 after hydrodynamic injection with NS or 20 μg pAAV/control plasmid. Results represent 2 independent experiments (*n* = at least 3/group)

We further detected the mRNA levels of cytokines and Th1-related immune factors in the liver MNCs 3 days AHI. No obvious difference in the mRNA levels of ifn-α, ifn-β, tgf-β, il-6, il-12 and il-15 was found between mice receiving NS or 20 μg of the control plasmids. However, the mRNA levels of Th1-related immune factors, including cxcr3 and t-bet, had a dramatic increase in 20μgof the control plasmid group. Also, interferon (IFN)-γ and IL-12 were slightly up-regulated (Fig. 7 and data not shown). These data suggested Th1 cells might play a critical role in HBV clearance caused by high doses of HBV plasmids in the TLRs independent manner.

## Discussion

A completely opposite immune responses toward HBsAg are induced in adult C57BL/6 mice after injection with 6 μg or 20 μg of pAAV/HBV1.2 plasmids. Our results have shown that HBV clearance still occurs in mice injected with20 μg of the e/core-null pAAV/HBV1.2 plasmids (Fig. 3c-d), and HBV persistence induced by 6 μg pAAV/HBV1.2 plasmid is broken by mixing with 14 μg of the control plasmid or with even other irrelevant plasmids at injection time (Fig. 4 and Fig. 5c). Therefore, the doses of plasmid backbone rather than the levels of HBV-related antigens played determining roles in triggering anti-HBV activity, resulting in the HBV clearance within 4 weeks AHI in our study.

Many components of HBV and plasmid backbones, including DNA from replication templates and RNA derived from transcriptional intermediate, are appropriate agonists of TLRs, RIG-I,NALP3, AIM2, IFI16 and so on [23, 24]. There are a large amount of innate immune cells [25] and accumulation of injected HBV plasmids AHI in the liver [7]. Therefore, the innate immune responses triggered by the plasmid DNA in the liver microenvironment were considered to be a critical factor in preventing HBV tolerance [26, 27]. Inflammatory response triggered by plasmid DNA should be induced both in the 6 μg and the 20 μg group, the opposite immune responses toward HBsAg induced by these two doses raise a possibility that the certain threshold for inflammatory response may exist to regulate the balance between liver tolerance and immune activation [28]. Furthermore, the mice kept in non-SPF conditions or old aged mice failed to establish the HBV persistence AHI with 6 μg pAAV/HBV1.2 (data not shown). Consistent with this observation, clinical data have shown that chronic HBV infection mostly occurs in childhood but only 10% of them appears in adults [29, 30].

Nevertheless, it seems hard to explain a very low HBV persistence observed in mice AHI with 1 μg low doses of pAAV/HBV1.2 plasmid [10]. Our previous studies revealed that HBV tolerance was mediated by HBsAg-specific Tr1 like cells and the time required for induction of these cells was at least 1 week [13, 17]. It is likely that the amount of HBsAg in mice receiving 1 μg pAAV/HBV1.2 plasmid is too little to induce HBV tolerance.

To explore the potential pathogen recogntion receptors (PRRs) that are responsible for HBV clearance induced by 20 μg plasmid DNA, we chose some TLR candidates that have interaction with DNA or RNA. Also, TLR4, a receptor recognizing bacterial liposaccarides, was tested to rule out the possibility that injected plasmid are mixed with LPS during plasmid DNA extraction in the present study. Our data suggest that TLRs signaling pathways are involved in suppressing HBV, but do not play a dominant role in final elimination of HBV caused by 20 μg HBV-containing plasmids, which is consistent with the report that serum HBsAg still disappear in Myd88^−/−^Trif^−/−^ mice at week 6 AHI [31]. The precise mechanisms of which PRRs involved in HBV clearance in our model need further investigation.

In our model, we found the mRNA levels of t-bet and cxcr3 were up-regulated significantly in mice injected with 20 μg pAAV/Control plasmids compared with mice injected with NS. T-bet is the transcription factor of Th1 cells and CXCR3 is the chemokine receptor expressed on Th1 cells [32]. These results further support a recent report that CD8^+^ Th1 cells played an important role in HBV clearance AHI with 20 μg pAAV/HBV1.2 plasmid [16].

## Conclusion

In this study we demonstrated that it is the dose of the plasmid backbone, but not HBV related antigens, regulates immune responses to determine HBV clearance in our mouse model. In addition, significantly increased mRNA levels of Th1-related immune factors teb-1, cxcr3 in the liver MNCs of mice receiving 20 μg of the plasmid backbone DNA suggest the involvement of Th1 cells in determination of HBV persistence or clearance.
